# In Vivo Raman Spectroscopy of Muscle Is Highly Sensitive for Detection of Healthy Muscle and Highly Specific for Detection of Disease

**DOI:** 10.1021/acs.analchem.4c03430

**Published:** 2024-09-26

**Authors:** James J.P. Alix, Maria Plesia, Daniel Stockholm, Pamela J. Shaw, Richard J. Mead, John C. C. Day

**Affiliations:** † Sheffield Institute for Translational Neuroscience, University of Sheffield, 385A Glossop Road, Sheffield S10 2HQ, United Kingdom; ‡ Neuroscience Institute, University of Sheffield, Western Bank, Sheffield S10 2TN, United Kingdom; § Généthon, Evry 91000, France; ∥ École Pratique des Hautes Études, PSL University, Paris 75000, France; ⊥ Interface Analysis Centre, HH Wills Physics Laboratory, University of Bristol, Tyndall Avenue, Bristol BS8 1TL, United Kingdom

## Abstract

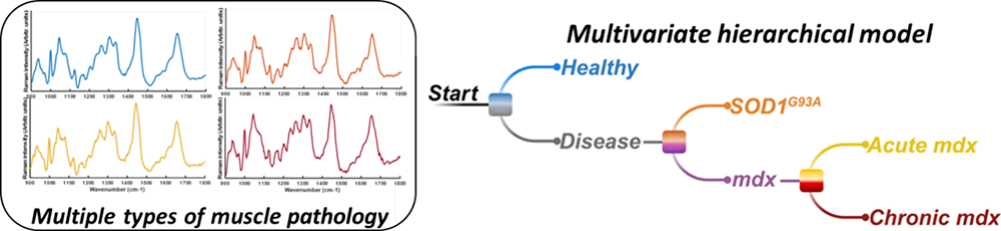

Raman spectroscopy of muscle provides a molecular fingerprint to identify the disease. Previous work has demonstrated effectiveness in differentiating between two groups of equal sizes (e.g., healthy vs disease) but imbalanced multiclass scenarios are more common in medicine. We performed in vivo Raman spectroscopy in a total of 151 mice across four different histopathologies (healthy, acute myopathy, chronic myopathy, neurogenic), with variable numbers in each (class “imbalance”). Using hierarchical modeling and synthetic data generation, we demonstrate high sensitivity (94%) for detection of healthy muscle and high specificity (≥97%) for disease. Further, we demonstrate the potential for unique biomarker development by demonstrating variations in the protein structure across different pathologies. The findings demonstrate the potential of Raman spectroscopy to provide accurate disease identification and unique molecular insights.

## Introduction

Raman spectroscopy is a new biomarker for the identification of disease-related changes in muscle. Through collection of inelastically scattered light, a highly detailed molecular fingerprint is obtained. This contains biochemical information, including, for example, information on protein folding, an increasingly recognized driver of neurological disease.^1^

Previously, we have shown that spontaneous Raman can be performed *in vivo* in preclinical models of neuromuscular disease.^2^ Analyses have shown promise in identifying different diseases in preclinical models of Duchenne muscular dystrophy (DMD) and amyotrophic lateral sclerosis (ALS). These have largely focused on the differentiation of two groups, typically healthy vs disease, or one pathology vs another (e.g., myopathy vs neurogenic), using well balanced data sets, i.e., roughly equal numbers in both groups.^2,3^ Similar analyses have been successfully undertaken using *ex vivo* human muscle samples.^4^

However, in clinical practice, physicians investigating patients for suspected neuromuscular disease rarely face a two-class problem. For example, a neurologist might form the hypothesis that a patient's weakness is due to a primary muscle disease, rather than a neurogenic cause, but there will then be several different myopathic disorders to consider.^5,6^ Furthermore, different diseases do not occur with equal incidence, so the frequency of classes in both the model training data and the test data may be skewed. This results in a class imbalance, which violates assumptions in many of the standard methods used to analyze Raman data.^7,8^ If Raman spectroscopy were to be deployed as a clinical tool in the context of neuromuscular disease, it would, as a minimum, be faced with healthy/neurogenic/myopathic class groups and class imbalance due to differences in the incidence of conditions within the neuromuscular disease spectrum.^9^

Herein, we test if, within a single analytical workflow, Raman spectroscopy can correctly identify four different types of muscle histopathology in preclinical models of muscle disease (DMD) and neurogenic disease (ALS): acute myopathy (early stage *mdx*), chronic myopathy (established *mdx*), neurogenic (SOD1^G93A^), and healthy. To explore the biochemical differences within these groups, we examined alterations in protein secondary structure. Our findings demonstrate that *in vivo* Raman spectroscopy of muscle generates a highly sensitive molecular fingerprint of healthy muscle and highly specific molecular fingerprints of disease, with differences in protein secondary structure.

## Experimental Section

### Preclinical Models

Experiments were conducted with University of Sheffield Ethical Review Sub-Committee approval and a UK Home Office (license number 70/8587), in accordance with the Animal (Scientific Procedures) Act 1986. The ARRIVE guidelines were followed.^10^

In the *mdx* model of DMD used, disease onset begins around 30 days with infiltration of inflammatory cells and myonecrosis, we have termed this stage “acute *mdx”*.^11^ This is followed by a more stable phase with regenerated, centrally nucleated myofibers; we studied mice aged 90 days and have termed these “chronic *mdx”*. In the SOD1^G93A^ model of ALS, hindlimb denervation begins at ∼40 days^12^; by the 90-day time point we studied, there is significant denervation and muscle atrophy.^13^ A healthy muscle group consisted of mice from the different genetic backgrounds of *mdx* and SOD1^G93A^. These included age-matched wild-type healthy control mice (C57BL/10ScSnOlaHsd; matched to *mdx*) and nontransgenic littermates from the SOD1^G93A^ colony (C57BL/6 J OlaHsd).

### Raman Spectroscopy

Fiber optic Raman spectroscopy was performed using a Raman probe housed within a 21-guage hypodermic needle. The incident light was provided by an 830 nm laser (power output 60 mW) and the probe was optically paired to the spectrometer for efficiency. The spectra were collected during a 40 s exposure.

The *in vivo* methodology was undertaken as previously described.^2^ Briefly, mice were anesthetized using 2% isoflurane. The fiber optic Raman probe was inserted into the medial and lateral heads of gastrocnemius bilaterally (collecting four spectra per mouse). We studied a total of *n* = 69 healthy mice and *n* = 89 disease mice (acute *mdx*: *n* = 24, chronic *mdx*: *n* = 39, 90-day SOD1^G93A^: *n* = 26), which provided an imbalance across some of the classes.

### Histology

Gastrocnemius muscles were dissected, snap-frozen, and cryosectioned at 10 μm. Haematoxylin and eosin staining was performed after warming to room temperature using a standard protocol.^14^ Slides were imaged using a digital slide scanner (Nanozoomer series, Hamamatsu).

### Data Analysis

Quantitative histological analysis of minimum Feret’s diameter and central nucleation was undertaken using the MyoSOTHES (Myofbers Segmentation wOrkfow Tuned for HE Staining) platform within QuPath/Cellpose.^15^ The variance coefficient (VC) of the minimum Feret’s diameter, a measure of the variation in muscle fiber diameter, was calculated as VC = (1000 × standard deviation of minimum Feret’s)/mean of minimum Feret’s. Quantification of the inflammatory cell infiltrate was performed within QuPath using the cell detection method.^16^ This was done by training a Random Forest classifier on a collection of inflammatory cells and then applying the model to whole sections. For all histological parameters, sections were analyzed from *n* = 3 mice for each of acute *mdx*, chronic *mdx*, and SOD1^G93A^ and *n* = 9 nontransgenic/wild type mice.

Raman analysis was performed using in-house code within MATLAB (2023a; MathWorks, USA) and the PLS Toolbox (Eigenvector Research Inc., USA). Spectra were interpolated and windowed in the “fingerprint region” (900 cm^–1^ to 1800 cm^–1^), where biologically relevant information is present. Outliers were removed using an algorithm to identify data more than three standard deviations outside the mean across a 15-wavenumber window. From a total of 604 spectra (4 spectra from each of 151 mice), 514 passed quality control for analysis (total of 151 mice). Background subtraction (an iterative asymmetric least-squares algorithm) and normalization (1-norm) on each spectrum were performed.

The data set was separated into training and test sets (Kennard Stone algorithm) with a 70:30 (training: test) split. The training data set thus comprised 143 spectra from *n* = 44 healthy mice, 54 spectra from *n* = 17 acute *mdx* mice, 106 spectra from *n* = 28 chronic *mdx* mice, and 55 spectra from *n* = 19 SOD1^G93A^ mice. The test data set comprised 66 spectra from *n* = 18 healthy mice, 26 spectra from *n* = 7 acute *mdx* mice, 43 spectra from *n* = 11 chronic *mdx* mice, and 21 spectra from *n* = 7 SOD1^G93A^ mice.

Using only the train data set, data were prepared for analysis using a hierarchical approach. The first step comprised identification of healthy (143 spectra, *n* = 44 mice) from disease (215 spectra, *n* = 64 mice) followed by separation of neurogenic (SOD1^G93A^; 55 spectra, *n* = 19 mice) from myopathic *mdx* (160 spectra, *n* = 45 mice) and then separation of acute (54 spectra, *n* = 17 mice) and chronic (106 spectra, *n* = 28 mice) *mdx*. Class imbalance, in which one class outnumbers another results in the accuracy paradox, can cause model overfitting and a failure to generalize to unseen data.^17^ To combat this, we generated synthetic data using the synthetic minority oversampling (SMOTE) algorithm.^18^ Thus, at each step, the two classes were balanced in number.

The balanced data sets were then processed for multivariate model generation. First, a generalized least-squares weighting multivariate filter was used to down-weight spectral features responsible for within-class variance. Mean centering was applied, and two-class partial least-squares discriminant analysis (PLS-DA) models were constructed with venetian blind cross-validation. Model complexity (latent variables [LV] number) was decided by viewing the cross-validation classification error against LV number plots, choosing the number of LVs at which the error plateaued. In addition, differences between the means plots were obtained by subtracting the mean of one group from the mean of another.

The hierarchical modeling function within the PLS Toolbox (Eigenvector Research, Inc., USA) then combined the individual models into a single workflow. The test data, which had not been seen during the model generation steps, was applied. Standard classification performance statistics were then calculated (accuracy (acc.), sensitivity (sens.), specificity (spec.), positive predictive value (PPV), negative predictive value (NPV), and *F* score..

**Table 1:** Hierarchical Model Performance Statistics on Unseen Test Data

	**acc.**	**sens.**	**spec.**	**PPV**	**NPV**	*F* score
healthy	0.84	0.94	0.77	0.74	0.95	0.83
acute mdx	0.92	0.61	0.98	0.89	0.93	0.73
chronic mdx	0.96	0.91	0.97	0.92	0.96	0.91
SOD1^G93A^	0.95	0.61	1	1	0.94	0.76

To gain insight into the most important spectral features used in the classifications, selectivity ratios were calculated.^19^ These were then multiplied by the sign of the relevant PLS-DA model regression vector so that values more important to the different classes could be visualized.^20^ This was done for both the training data and the test data. A simplified representation of key spectral regions at each step in the workflow was generated by identifying the spectral regions with high discriminating value important wavenumbers by an F-test (probability level α of 0.95). The important regions were identified in each binary model and aggregated into a “block plot” to provide a simple visualization.

Determination of protein secondary structure was undertaken using Origin (2023).^21^ Correctly predicted spectra from the test group were identified, and the amide I region (1590–1720 cm^–1^) was separated from the rest of the spectrum. Group means for the healthy, acute *mdx*, chronic *mdx*, and SOD1^G93A^ groups were generated and then scaled between 0 and 1. A mixed Lorentz–Gaussian (Voigt) function was used. Six peaks centered on 1601 and 1615 cm^–1^ (aromatic amino side chains), 1635 cm^–1^ (nonregular), 1652 cm^–1^ (α-helix), 1663 cm^–1^ (β-sheet), and 1677 cm^–1^ (nonregular) were used for fitting, with the addition of two further peaks (1700 and 1710 cm^–1^, nonregular) in chronic *mdx*. The starting height for each peak was the amide I spectral intensity at that wavenumber. The full width at half-maximum was enabled to an upper limit of 30 cm^–1^. The proportion of the aromatic amino acid and secondary structure components was then reported as the percentage of the given peak relative to all peaks utilized in the fitting. A more complex secondary structure analysis was also performed utilizing matrix factorization to obtain information at the level of individual spectra (see supplemental methods for details).^21^

## Results and Discussion

### Raman Spectra and Quantitative Histopathology

**Figure 1:**
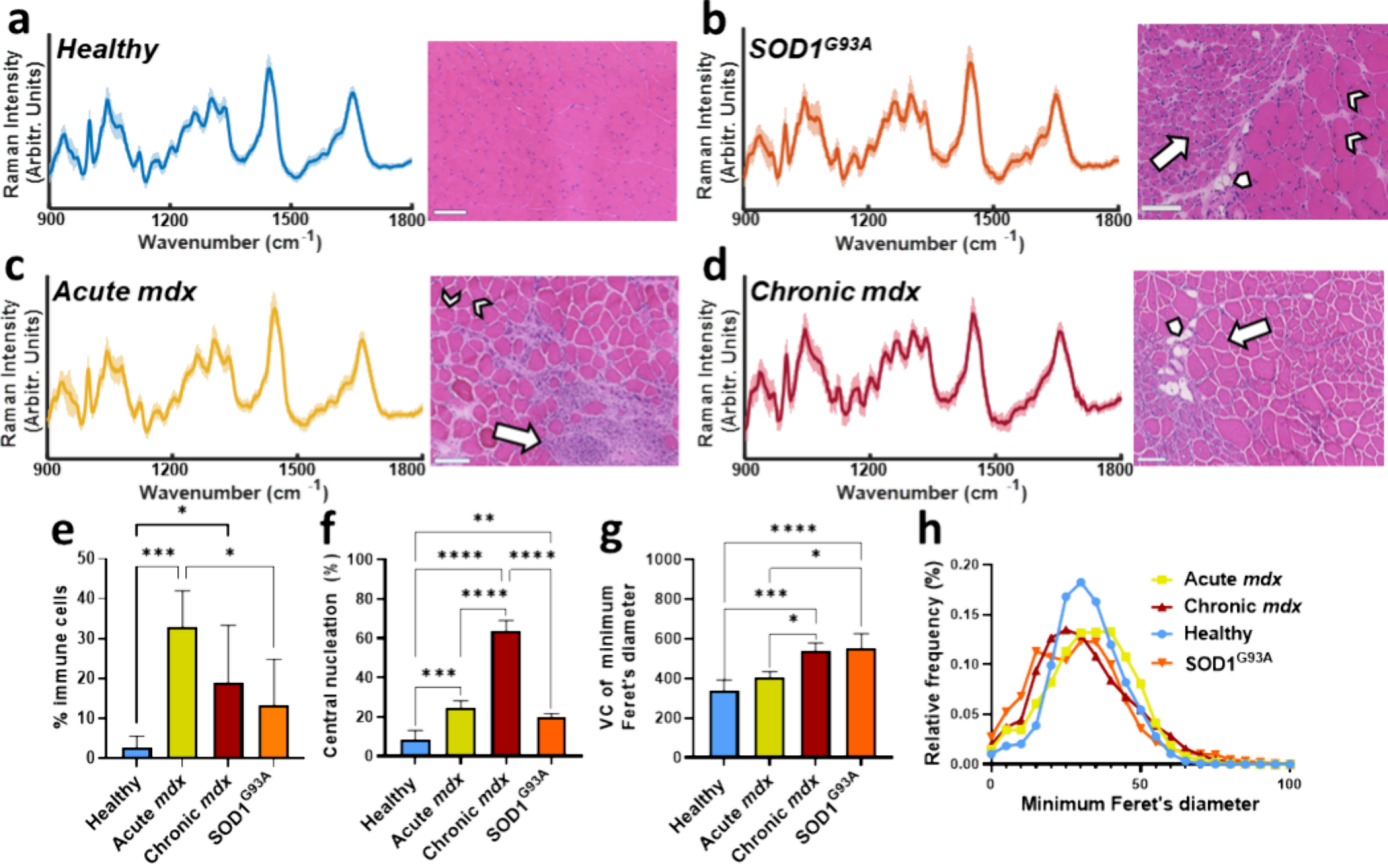
*In vivo* Raman spectra and histology from the four groups studied. (a) Healthy (average of 143 spectra from 44 mice). Healthy muscle is characterized by muscle cells of similar size with their nuclei at the periphery of the cell. (b) SOD1^G93A^ (average of 55 spectra from 19 mice). SOD1G93A muscle shows grouped atrophy (collections of smaller cells, arrow) and some fat deposition (arrowhead), together with some normal cells (chevrons). (c) Acute *mdx* (average of 54 spectra from 17 mice). The muscle manifests inflammatory cell infiltrates (arrows), together with healthy muscle cells (chevrons). (d) Chronic *mdx*, (average of 106 spectra from 28 mice). Chronic *mdx* demonstrates fat deposition (arrowhead), with centrally nucleated regenerated muscle cells (arrow). (e–h) Quantitative histopathology for the four conditions (healthy *n* = 9 mice; acute *mdxn* = 3 mice; chronic *mdxn* = 3 mice; SOD1^G93A^*n* = 3 mice). VC – variation coefficient (a measure of muscle fiber size variation). For simplicity, only significant comparisons are shown. Scale bars 100 μm. *p < 0.05, ***p < 0.001, ****p < 0.0001.

*In vivo* Raman spectra were obtained, and muscle was studied histologically (Figure 1). The spectra all contained similar core features, such as phenylalanine (1000 cm^–1^), the CH_2_ deformation of proteins/lipids (1450 cm^–1^), and the amide I protein peak (1650 cm^–1^; Figure 1). Histology of healthy tissue demonstrated peripherally nucleated myofibers of similar size. Histology from SOD1^G93A^ mice demonstrated grouped atrophy and normal-appearing fibers. Acute *mdx* muscle showed inflammatory cells (typically reported to be macrophages, CD4+, and CD8+ T lymphocytes),^22^ while the chronic *mdx* muscle was dominated by regenerated, centrally nucleated fibers, variation in muscle fiber size, and some evidence of fat deposition.

Thus, the chosen models manifested quantitatively different histopathology. Furthermore, the artificial intelligence-driven MyoSOTHES workflow aligns well with the prior literature using more traditional methods. For example, Massopust et al. found that in the *mdx* hindlimb, ∼20% of fibers had central nuclei at 42 days,^23^ while results at 90 days onward ranged from 65 to 90%.^23,24^ In SOD1^G93A^ at 90 days, motor neurone counts in the lumbar spinal cord are around half that of wild-type mice,^12,25^ and muscle fiber size variation in gastrocnemius is apparent.^26^

### Hierarchical Classification Model

**Figure 2:**
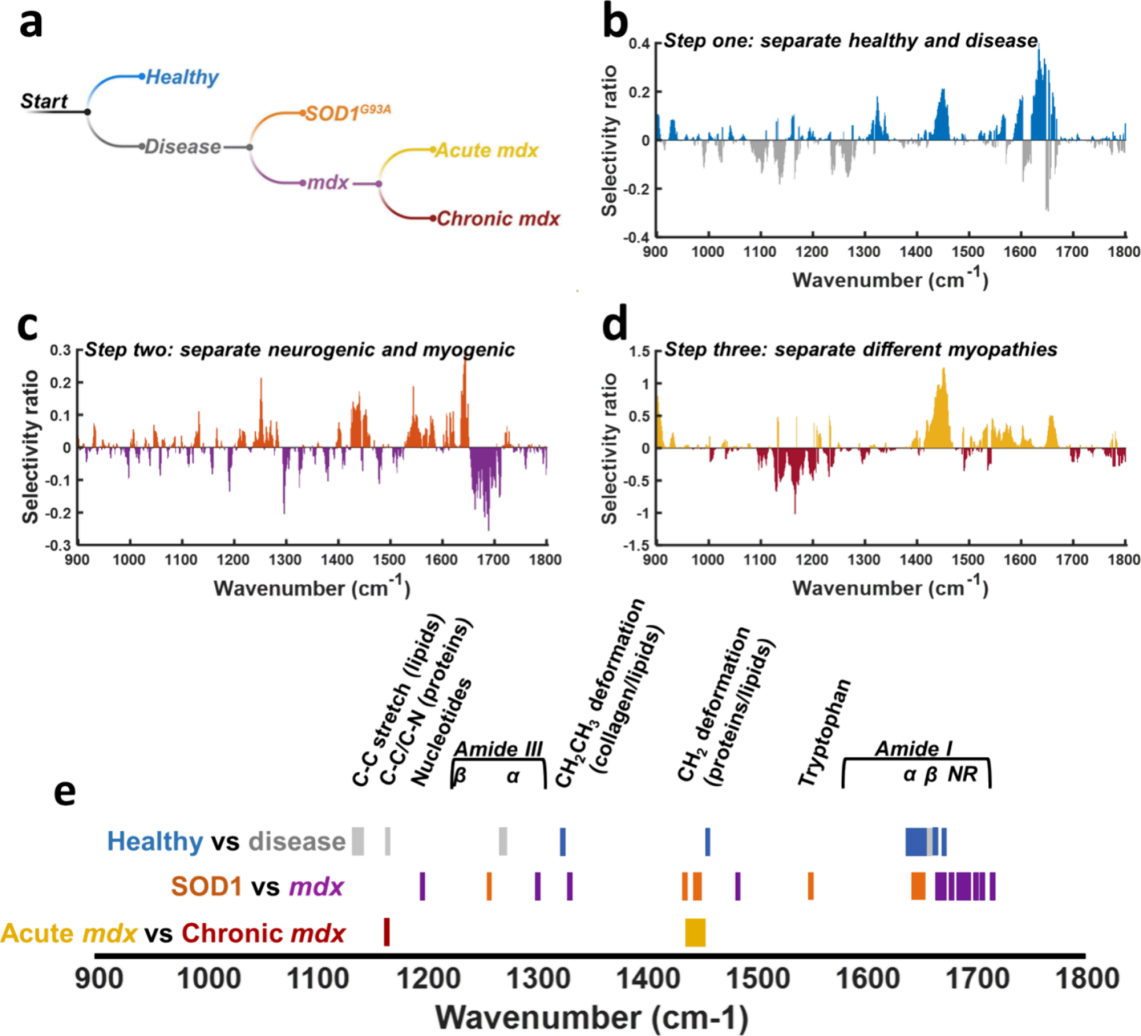
The hierarchical model and important spectral features in each step. (a) The hierarchical model workflow. (b–d) Selectivity ratio plots. These record the importance of each spectral feature at each of the steps in the hierarchical model. More important features result in higher scores. (e) To visualize important spectral regions across the steps in the hierarchical model, a block plot is created using features with high selectivity ratio scores.

To classify the four groups, a hierarchical decision tree was constructed (Figure 2). At each step, a binary model node removed one class. The LVs underpinning the model, together with model performance, can be seen in Figures S1–S3. The first step separated healthy muscle from a combined group of “disease” (acute and chronic *mdx* and SOD1^G93A^). The second step removed SOD1^G93A^ from a combined myopathy group (acute/chronic *mdx*) and, finally, acute and chronic *mdx* were separated. These models were then combined into a single workflow.

At each step, selectivity ratio plots were constructed to aid interpretation, and spectral regions particularly useful for discrimination at each step were aggregated into a block plot. This demonstrated that differences in protein and lipid biochemistry were driving class distinctions, with particularly prominent areas including the CH_2_ deformation of proteins and lipids (∼1450 cm^–1^) and the amide I region (1590–1720 cm^–1^). Similar regions of importance were also identified in the latent variable loadings plots (Figures S1–S3) and differences between the means plots (Figure S4). Similar spectral features have also been reported in human muscle disease^4^ and in fly models of muscle disease.^27^

The hierarchical approach is analogous to the diagnostic process of expert clinicians who rapidly generate hypotheses that are then systematically evaluated.^28^ In neuromuscular neurology, a simple example might be deciding through clinical history and examination that a patient’s weakness is neurological in origin, versus, for example, musculoskeletal. Next, the hypothesis that the problem is myopathic in origin may be interrogated versus, for example, a motor neurone etiology. This would first be evaluated through the clinical history and examination and then through combinations of electromyography (EMG), MRI, blood tests, and muscle biopsy.^29^ Thus, our approach aligns with clinical decision-making by first identifying disease and then separating different pathologies. However, just as the diagnostic decision-making may proceed in alternative ways, our Raman workflow is not the only way that the data could be manipulated. For example, a clinician may determine very quickly that a case is neurogenic in origin, and thus, a mirroring Raman step would be the separation of neurogenic pathology from a combination of healthy and myogenic. Within the analysis algorithm, a prior probability weighting could also be added taking into account epidemiology and/or expert clinical opinion.^30^

A test data set, kept separate and not used in any of the model steps, was then fed into the hierarchical model. The results demonstrated a highly sensitive detection of healthy tissue, with highly specific detection of the different pathologies (Tables 1 and S1). This pattern can be appreciated in histology, where some relatively normal histological areas are encountered in acute *mdx* and SOD1^G93A^.

In the current analysis, we have focused on a standard analysis algorithm in Raman spectroscopy, PLS-DA. However, alternative techniques, particularly nonlinear or deep learning approaches, may provide superior performance, particularly with a greater number of samples.^31^ In the current analysis, we have utilized the whole spectrum, but some authors suggest that selection of key features, either through domain-specific knowledge or algorithmic feature selection techniques, can improve classification.^32^ Furthermore, targeting the Raman signal to specific areas within the muscle may also increase the diagnostic yield. The best way to do this is unknown at present, although our recently described combination of EMG and Raman (“optical EMG”)^13^ could permit real-time targeting via EMG signal interpretation and a unique “electro-photonic” assessment of muscle health. Alternative bedside methods such as neuromuscular ultrasound may also be worth exploring.^33^

### Protein Secondary Structure Analysis

**Figure 3:**
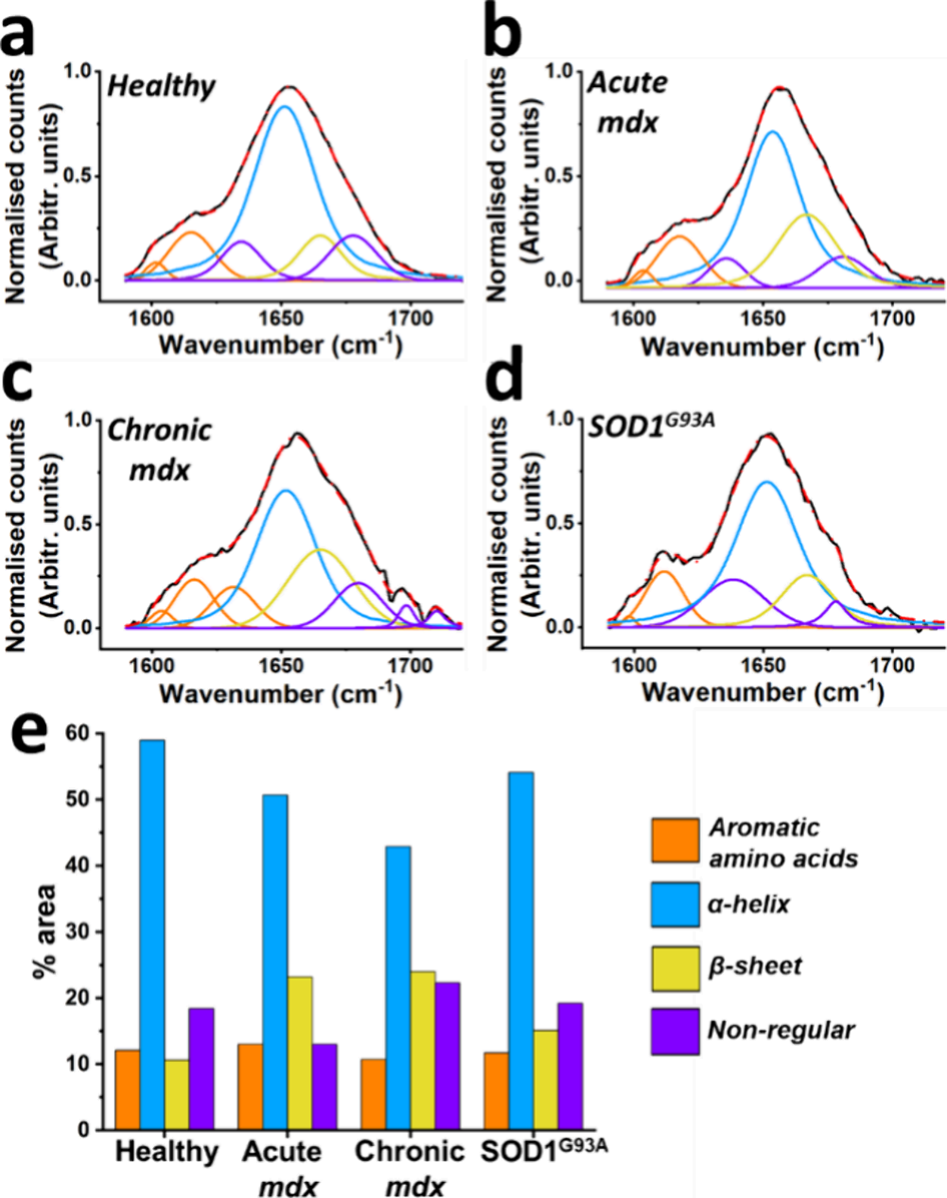
Protein secondary structure in myopathic and neurogenic muscle. (a–d) The amide I region (1590–1720 cm^–1^) was selected from the Raman spectra of the correctly predicted test spectra. A group average was created and amide I peak fitting performed. A simple visual inspection of the fitted peaks reveals, for example, an increase in the β-sheet related peak (yellow). (e) Differences in the contribution of aromatic amino acids and different protein secondary structure conformations are shown as a percentage of the total area. While α-helices are the dominant structure across all four groups, increases in β-sheet and nonregular structures seen in disease, particularly in the myopathy groups (acute and chronic *mdx*).

In the block plot (Figure 2e), important features are consistently seen in the amide I region. This part of the Raman spectrum is useful for investigating protein secondary structure conformations^34^ and so can provide a unique insight into the biochemistry of the muscle. To explore this further, spectra in the test data set that were correctly allocated to their group (healthy/acute *mdx*/chronic *mdx*/SOD1^G93A^) were chosen for further amide I analysis. Peak fitting for different structures (and aromatic amino acids) demonstrated that healthy muscle had a large abundance of α-helix structures (Figure 3). Acute *mdx* muscle demonstrated an increase in β-sheet, while chronic *mdx* manifested large increases in both β-sheet and nonregular conformations. SOD1^G93A^ was characterized by a modest change in β-sheet. These changes were assessed at the group level, and so information on the importance of different conformations to individual spectra is not available. This can be achieved using matrix factorization, which provides information at the level of individual spectra that is amenable to statistical testing (supplemental methods and Figure S5).

When considering the changes to secondary protein structure, it is important to note that by taking Raman spectra from intact muscle, we are studying protein structure at the level of the whole muscle. We therefore caution against an interpretation based on individual proteins. Notwithstanding this, misfolded proteins are a known occurrence in many neurological conditions, including muscle disorders,^35,36^ where they may potentiate already abnormal physiology.^37−39^ The most marked changes in protein structure were evident in *mdx* mice, a model of Duchenne muscular dystrophy. In this disease, impaired autophagy is proposed to limit the removal of protein aggregates^40^ and this has been reported in *mdx*.^41^ Thus, one possibility is that abnormal protein conformations relate to this defect. Alternatively, they might relate to a more nonspecific response to muscle necrosis,^39^ atrophy,^42^ and/or regeneration.^43^

## Conclusions

We have demonstrated that Raman spectroscopy can effectively identify healthy and diseased muscle in a complex, imbalanced multiclass setting. Biochemical interpretation demonstrated significant changes in the protein structure. The analysis provides a platform for further development of clinical Raman spectroscopy in neuromuscular disease.

## Supporting Information Available

Figures S1–4 and Table S1; LVs and performance of the models in hierarchical workflow; methods and data for non-negative matrix factorization of amide I profiles (PDF)
