# Supplementary material for: In Vivo Raman Spectroscopy of Muscle Is Highly Sensitive for Detection of Healthy Muscle and Highly Specific for Detection of Disease
Source: Anal Chem. 2024 Sep 26;96(40):15991–7. doi: 10.1021/acs.analchem.4c03430 (PMC11465232; doi:10.1021/acs.analchem.4c03430)
Supplement: Supplementary file 1 — ac4c03430_si_001.pdf [file ac4c03430_si_001.pdf]

Supporting information for:

In vivo Raman spectroscopy of muscle is highly sensitive for detection of healthy muscle and highly specific for detection of disease

James J.P. Alix[a,b*], Maria Plesia[a], Daniel Stockholm[c,d], Pamela J. Shaw[a,b], Richard J. Mead[a,b], John C. C. Day[e]

[a]Sheffield Institute for Translational Neuroscience, University of Sheffield.

[b]Neuroscience Institute, University of Sheffield

[c]Généthon, 91000, Evry, France.

[d]École Pratique des Hautes Études, PSL University, 75000, Paris, France

[e]Interface Analysis Centre, School of Physics, University of Bristol

*Email: j.alix@sheffield.ac.uk

Table of contents

Supplemental methods S3

Figure S1. Latent variable information (loadings and scores) and ROC plots for the model healthy vs. disease. S4

Figure S2. Latent variable information (loadings and scores) and ROC plots for the model $SOD1^{G93A}$ vs. myopathy. S5

Figure S3. Latent variable information (loadings and scores) and ROC plots for the model acute myopathy vs. chronic myopathy. S6

Figure S4. The difference between the means plots for the steps within the hierarchical model. S7

Supplemental table 1. Confusion matrix for the test data set predictions. S7

Figure S5. Non-negative matrix factorisation derived spectral patterns show conformational differences in protein secondary structure. S8

Supplemental references S10

Supplemental methods

To aid evaluation of protein conformations at the level of individual spectra a matrix factorisation-based analysis was performed.[1] In this, spectral patterns were obtained through a hierarchical alternating least squares non-negative matrix factorisation (NMF) algorithm optimised for low rank solutions.[2] NMF approximates the original data (A, an n x m matrix where, in this analysis, n is the number of samples and m is spectral wavenumbers) as the product of two lower rank matrices, A = WH, where W represents the derived spectral patterns and the matrix H represents the relative importance (the weights) of those patterns to each sample.

To estimate the relative contributions of different secondary structures within each of the calculated patterns, the second derivative of the was calculated and subjected to a Savitzky-Golay smooth (second order, 5 data points). Peaks were then identified using a 20% threshold, which excluded minor peaks, and a Voigt fitting function utilised. Peaks were allocated to α-helix (1650-1658 cm^{-1}), β-sheet (1664-1673 cm^{-1}) and nonregular (1630-1640, 1674-1689 and 1700-1710 cm^{-1}) structures.

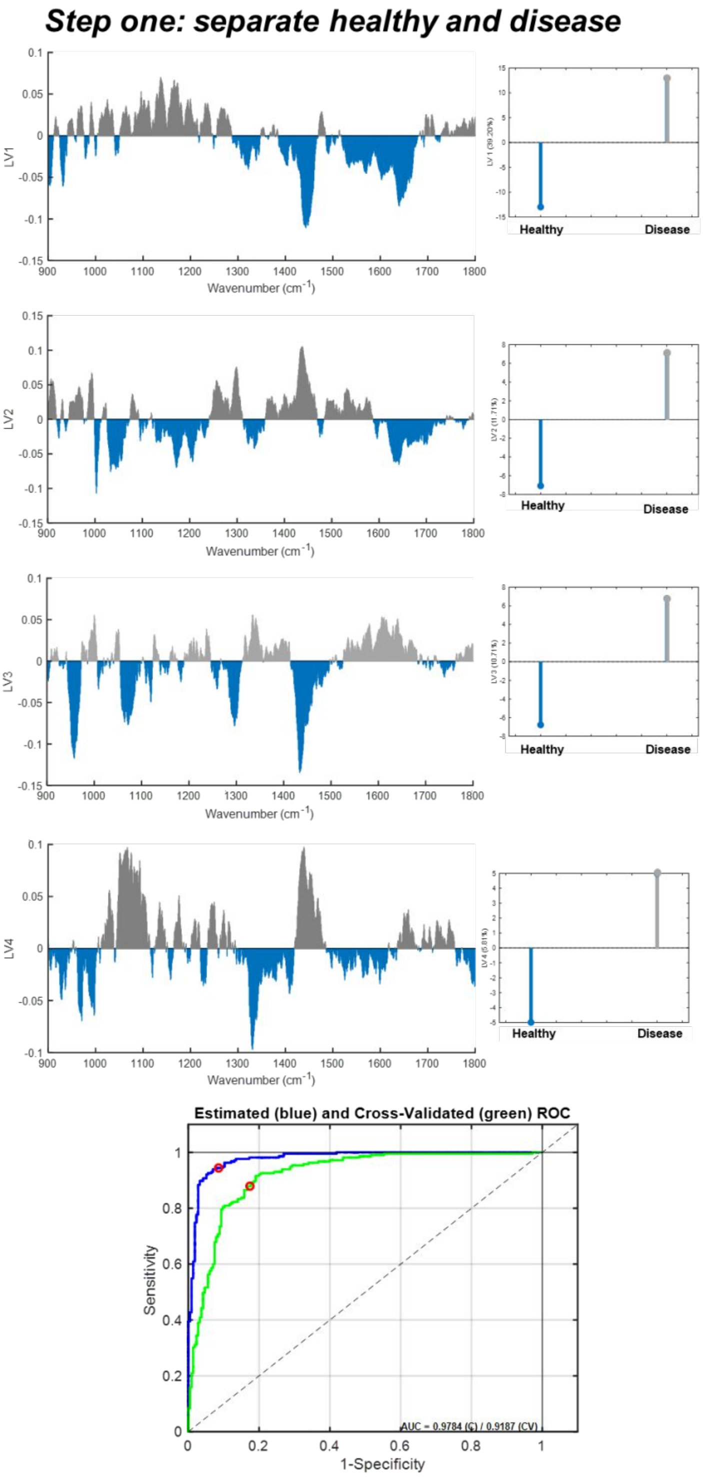

Figure S1. Latent variable information (loadings and scores) and ROC plots for the model healthy vs. disease.

The latent variable loadings plots provide information on the importance of different wavenumbers to the model. The scores plots then demonstrate which class different spectral regions are important to. In the above, spectral regions with negative values are important to the healthy group. The receiver operating characteristic (ROC) curve is also shown and displays the calibrated (C) and cross validated (cv) model performance.

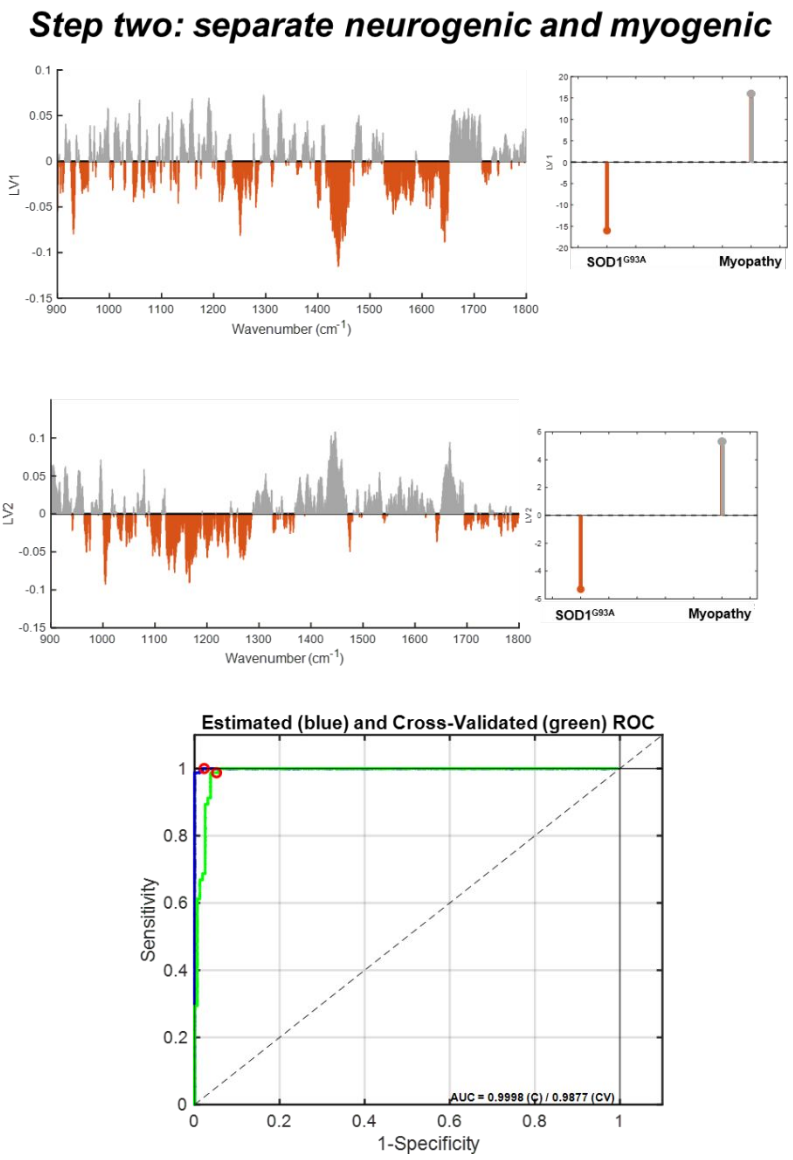

Figure S2. Latent variable information (loadings and scores) and ROC plots for the model SOD1^{G93A} vs. myopathy.

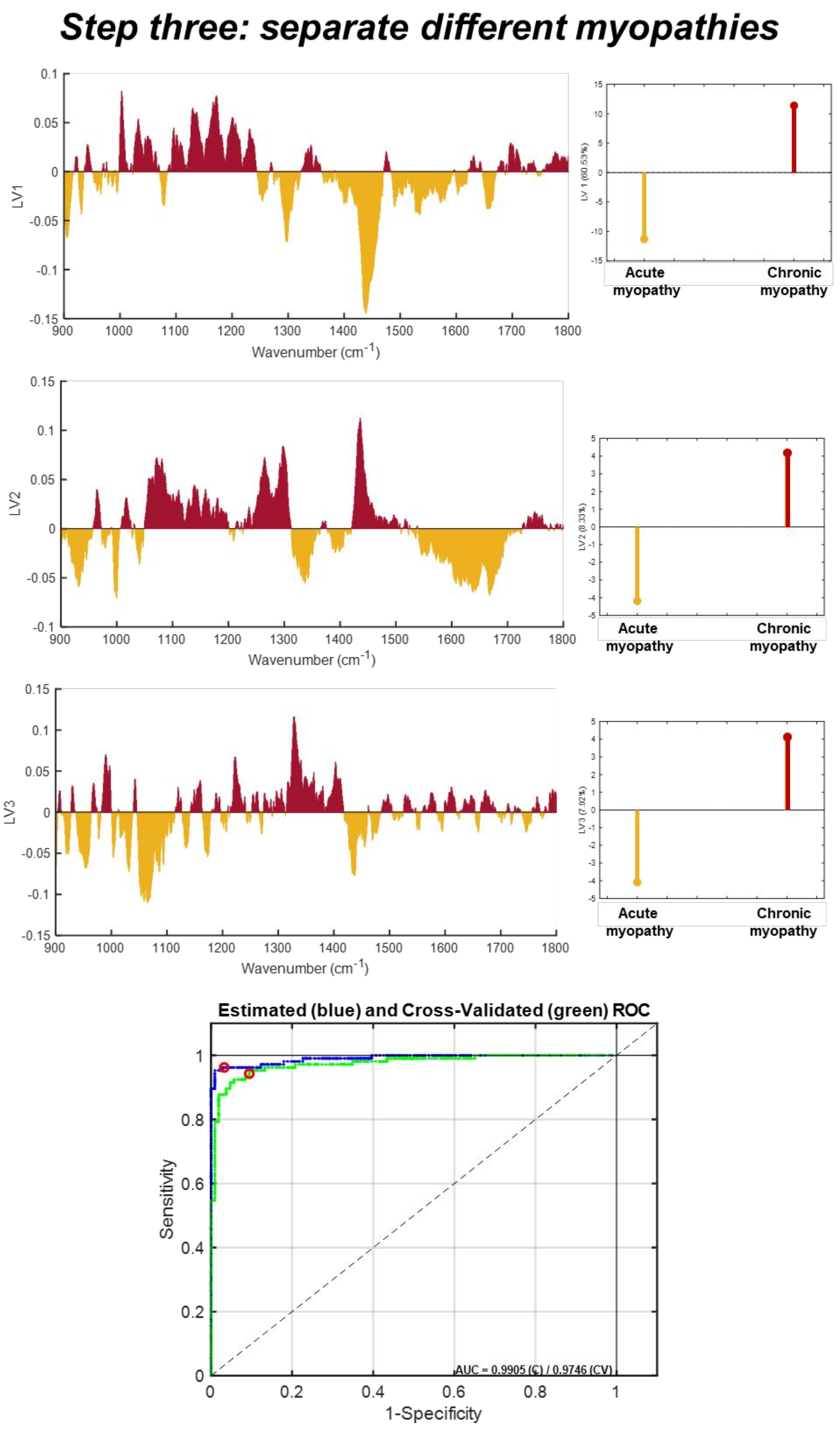

Figure S3. Latent variable information (loadings and scores) and ROC plots for the model acute myopathy vs. chronic myopathy.

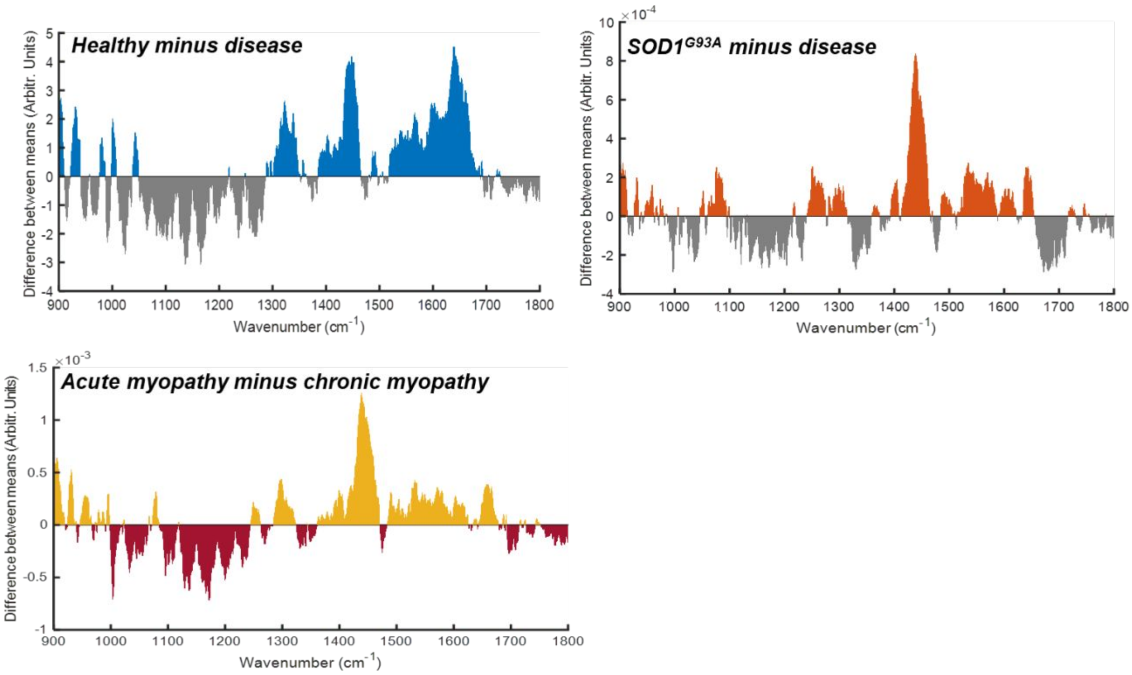

Figure S4. The difference between the means plots for the steps within the hierarchical model.

TRUE CLASS \ PREDICTED CLASS	Healthy	Acute myopathy	Chronic myopathy	SOD1^{G93A}
Healthy	62	1	3	
Acute myopathy	10	16		
Chronic myopathy	3	1	39	
SOD1^{G93A}	8			13

Supplemental table 1. Confusion matrix for the test data set predictions.

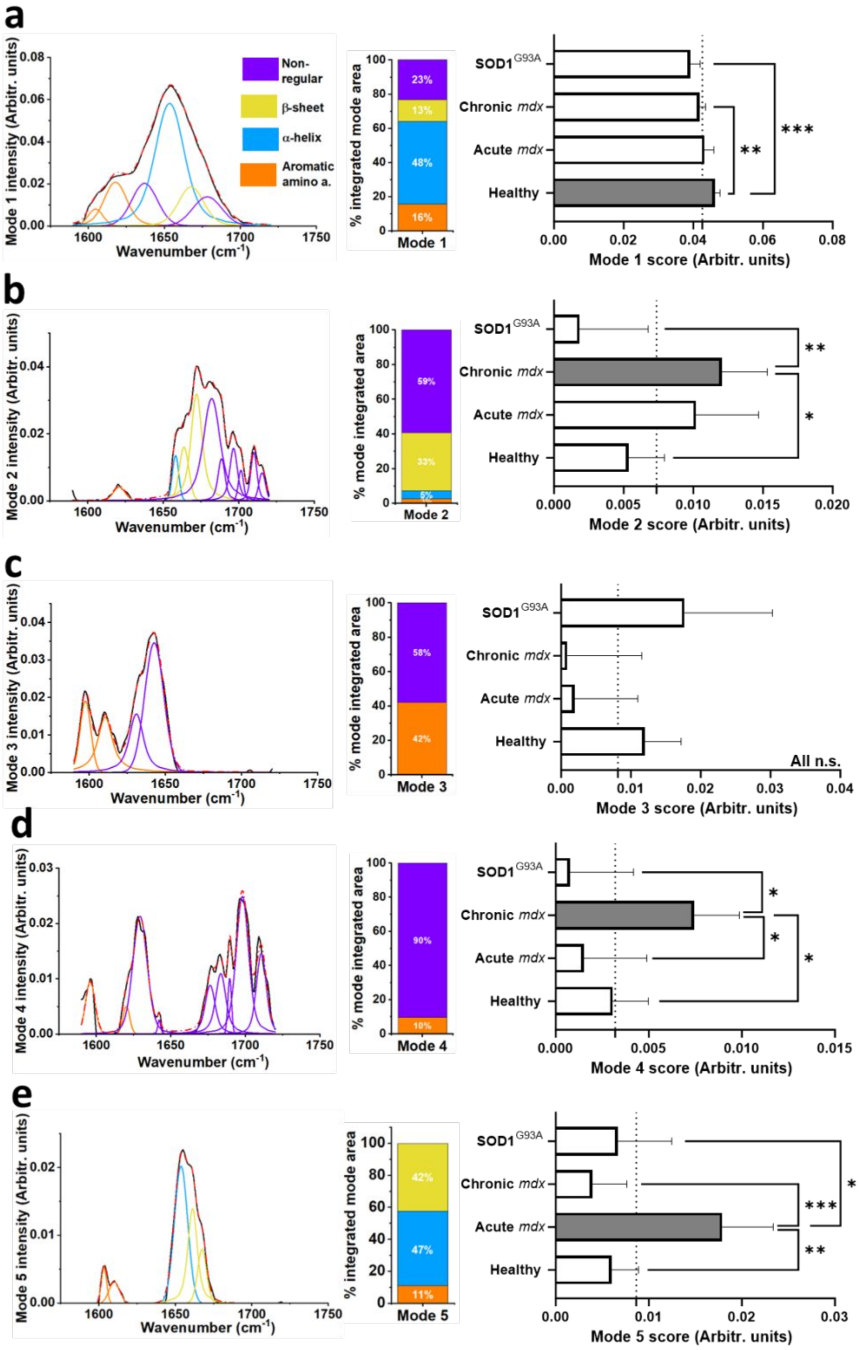

Figure S5. Non-negative matrix factorisation derived spectral patterns show conformational differences in protein secondary structure.

Using amide I spectra, non-negative matrix factorisation was performed. Five unique spectral patterns were obtained (A-D, these are termed 'modes'). Peak fitting profiles are shown within the pattern profiles.

The integrated area for each protein structure is then shown as a stacked bar chart, allowing clear differences in conformational content to be appreciated. The mode scores, which represent the importance of a given mode to each sample, were then subjected to statistical testing.

Mode 1 (shown in (a)) had an overall profile more prominent in healthy muscle (and acute *mdx*). Modes 2 and 4, were more prominent in chronic *mdx*, while mode 5 was more prominent in acute *mdx*. *P<0.05, **P<0.01, ***P<0.001.

Supplemental references

1. Alix JJP, Plesia M, Dudgeon AP, et al. Conformational fingerprinting with Raman spectroscopy reveals protein structure as a translational biomarker of muscle pathology. Analyst. 2024 Apr 29;149(9):2738-46.
2. Atif SM, Qazi S, Gillis N. Improved SVD-based initialization for nonnegative matrix factorization using low-rank correction. Pattern Recognition Letters. 2019 2019/05/01/;122:53-9.
